# Attenuated maladaptive emotion processing as a potential mediator of the relationship between dispositional mindfulness and mental health

**DOI:** 10.1016/j.heliyon.2023.e21934

**Published:** 2023-11-02

**Authors:** Rakesh Pandey, Satchit Prasun Mandal, Meenakshi Shukla, Vishnukant Tripathi, Elena Antonova, Veena Kumari

**Affiliations:** a Department of Psychology, Banaras Hindu University, Varanasi, India; b Department of Psychology, Rajiv Gandhi University, Rono Hills, India; c Department of Psychology, University of Allahabad, Prayagraj, India; d Division of Psychology, Department of Life Sciences, College of Health, Medicine and Life Sciences, Brunel University London, Uxbridge, UK; e Centre for Cognitive and Clinical Neuroscience, College of Health, Medicine and Life Sciences, Brunel University London, Uxbridge, UK

**Keywords:** Dispositional mindfulness, Mental health, Maladaptive emotion processing, Adaptive emotion processing, Negative affect, Positive affect, Emotional pathways

## Abstract

The emotion processing and regulation mechanisms by which dispositional (personality trait) mindfulness exerts its positive effects on mental health remain unclear. Here, we tested, using structural equation modeling, whether the relationship between higher dispositional mindfulness and better mental health is mediated by reduced maladaptive processing of emotional information (e.g., expressive suppression, impoverished emotional experiences, unprocessed emotions, avoidance, externalizing strategies) and associated lower negative affect, enhanced adaptive processing of emotional information (e.g., cognitive reappraisal) and associated higher positive affect, or a combination of these two emotion processing styles. Dispositional mindfulness, mental health, diverse emotional constructs with adaptive and maladaptive dimensions (including range and differentiation of emotional experiences, use of specific emotion regulation strategies, emotion processing deficits, negative affect repair strategies, negative mood regulation expectancies), and positive and negative affect were assessed using self-report measures in a non-clinical sample of 256 adults. The relationship between higher dispositional mindfulness and better mental health was found to be best explained by reduced maladaptive emotion processing styles and associated lower negative affect, rather than by enhanced adaptive emotion processing and higher positive affect. Further research should investigate whether the same mechanisms explain psychological benefits of cultivated mindfulness in people with low dispositional mindfulness and/or with mental health disorders following mindfulness skills training.

## 1 Introduction

Mindfulness refers to a non-judgmental and non-reactive awareness of internal (e.g., thoughts, physical sensations) as well as external (e.g., sounds, smells) experiences of the present moment [1]. Although mindfulness has been associated with a form of Buddhist meditation practice [2], its personality trait-like qualities displayed in varying degrees by meditation-naïve individuals have also been recognized and commonly referred to as ‘dispositional mindfulness’ (DM) [3]. There is evidence that DM may exist independently of learnt or cultivated (through training) mindfulness as an innate individual difference in experiencing mindful awareness and expressing it behaviourally in everyday life [4].

DM is characterized by the same two main components as cultivated mindfulness [4], captured by the operational definition of mindfulness: i) the self-regulation of attention maintained on the present-moment experience; and ii) adopting a particular orientation towards the present-moment experience, characterized by curiosity, openness, and acceptance [5]. Both components of the operational definition are important in the context of emotion regulation and well-being. The self-regulation of attention (first component) should promote a non-elaborative present-moment awareness of the thoughts, feelings, and sensations, instead of being caught up in them and ruminating about their origins or implications [6]. The experiential orientation (second component) of acceptance should improve affect tolerance, whilst openness should reduce experiential avoidance, promoting receptivity to experiences [5]. It should also facilitate an insight that the thoughts and feelings are passing events in the mind rather than valid reflections of reality or aspects of self [6] – this is akin to the concept of *decentering* in Cognitive-Behavioral Therapy (CBT), which has been linked to the CBT's efficacy in depression-relapse prevention [7].

Indeed, mindfulness-based interventions (MBIs) have been reported to reduce symptoms and enhance well-being in patients with psychological disorders [8,9], whilst DM has been found to have inverse relationships with symptoms of psychological disorders, such as anxiety and depression [10] in nonclinical populations. Furthermore, DM measured by self-report has been found to inversely associate with neuroticism [4,11], a personality trait also referred to as negative affectivity [12], emotional instability [13] and negative emotionality [14], and is known to reduce the risk for depression and anxiety disorders [15,16]. Conversely, self-reported DM correlates positively with extraversion, specifically its aspects that are related to the ability to experience positive affect rather than to high arousal or drive [4].

The precise mechanisms through which trait mindfulness promotes mental health and well-being remain unclear. Different mechanisms of mindfulness meditation and MBIs within the context of emotion regulation strategies have been proposed [[17], [18], [19], [20], [21], [22]]. There is a relative theoretical consensus that trait mindfulness, whether trained or dispositional, is characterized by a reduction of maladaptive emotional processes, with the evidence for inverse relationships of trait mindfulness with thought suppression [23], expressive suppression [24], and experiential avoidance [25]. However, there has been much debate as to whether cognitive reappraisal, an adaptive emotional process, is an aspect of mindful emotion regulation [20]. Whereas some researchers include cognitive reappraisal amongst the integral mechanisms of mental health improvements as a result of mindful meditation practice and MBIs (e.g., [19,22]), others note that cognitive reappraisal is not an inherent goal of mindfulness practice (e.g., [18]) since cognitive reappraisal involves actively reinterpreting emotional stimuli in a way that modifies their emotional impact [26], whilst mindfulness, as secularly defined, involves experiencing mental events, including emotions, without explicitly manipulating them in some way [27]. It has also been proposed that cognitive reappraisal might only be important for the novice mindfulness practitioners, but it is not a mechanism of mindful emotion regulation in long-term mindfulness practitioners [17]. In the context of this debate, the interplay between DM and cognitive reappraisal in relation to mental health and well-being is even less clear.

The present study therefore investigated the unique and/or differential roles of both maladaptive (e.g., expressive suppression, impoverished emotional experiences, unprocessed emotions, avoidance, externalizing strategies) and adaptive (e.g., cognitive reappraisal) emotional processing styles in DM mental health relationship with two broad aims. First, we aimed to comprehensively examine the relationship between DM and a range of mental health dimensions as well as emotional constructs, expecting DM to be associated positively with mental health and well-being, and to correlate positively with adaptive, and negatively with maladaptive, dimensions of various emotional constructs. Our second, and more important, aim was to comprehensively test whether DM is associated with mental health primarily through the mediating role(s) of attenuated maladaptive emotion processing and negative affect (NA), enhanced adaptive emotion processing and positive affect (PA), or a combination of these two pathways.

## 2 Methods

### 2.1 Participants

The study involved 256 adults (144 males, mean age = 23.04 ± 2.88 years; 112 females, mean age = 22.36 ± 2.01 years) from different post-graduate colleges and universities in Varanasi, India. The participants self-reported belonging to middle socioeconomic status, had no history of mental or physical illnesses, were not on regular medication, not regularly using cigarettes or alcohol (<5 times a month), and also had no history of substance abuse. Our sample size accommodates mild-to-moderate effect sizes for correlations of interest and allows a variables-to-participant ratio of 1:10 for factor analysis [28].

The study protocol and procedures were approved by the University Research Programme and Departmental Research Committee, Department of Psychology, Banaras Hindu University, India (Ref: Psych./Res./September 2010/). All participants provided written informed consent prior to their participation.

### 2.2 Self-report measures

The following measures (for further details, see Supplementary Table 1) were used.

#### 2.2.1 Dispositional mindfulness (DM)

The Hindi adaptation of the Five-Facet Mindfulness Questionnaire (FFMQ-H) [29] was used. It has 28-items and assesses four facets: *Describing* (8 items), *Acting with Awareness* (8 items), *Non-judging* (7 items), and *Non-reactivity* (5 items). The scale does not include *Observing* as it is found to exist only in experienced meditators [30]. Higher scores indicate greater DM. In the present sample, the questionnaire showed acceptable-to-good internal consistency (Cronbach's alpha coefficient for the full scale: .80; and for *Describing*, *Acting with Awareness*, *Non-judging*, and *Non-reactivity* subscales: .79, .82, .77, and .60, respectively).

#### 2.2.2 Mental health

The Hindi adaptation of the 90-item Revised Symptom Checklist (SCL-90-R-H) [31] was used. It assesses nine primary symptom dimensions: *Somatization*, *Obsessive-compulsive*, *Interpersonal sensitivity*, *Depression*, *Anxiety*, *Hostility*, *Phobic anxiety*, *Paranoid ideation*, and *Psychoticism*, and also provides three global indices: (1) *Positive Symptom Distress Index* (PSDI; a pure intensity measure of mental health problems), (2) *Positive Symptoms Total* (PST; the total number of self-reported symptoms), and (3) *Global Severity Index* (GSI; the average score of the 90 items, considered the single best indicator of overall distress level and psychopathology). In the current sample, Cronbach's alpha coefficient for the full scale was .98.

#### 2.2.3 Emotional constructs

##### 2.2.3.1 Range and differentiation of emotional experiences

We used the Hindi version of Range and Differentiation of Emotional Experience Scale (RDEES-H) [29], a 14-item measure with two dimensions: *Range* (ability to experience a wide range of emotions; 7 items) and *Differentiation* (capacity to draw subtle distinctions among the felt emotional experiences; 7 items). Each item requires responding on a 7-point Likert scale (1 = ‘not at all characteristic’ to 7 = ‘extremely characteristic’). Higher scores indicate more varied and diversified emotional experience. In the current sample, the internal consistency of the overall scale was .80, and that of *Range* and *Differentiation* dimensions was .60 and .82, respectively.

##### 2.2.3.2 Emotion regulation strategies

The Hindi version of the 10-item Emotion Regulation Questionnaire (ERQ) [32] was used to assess the habitual use of emotion regulation strategies: *Cognitive Reappraisal* (reinterpreting an emotion-arousing event in a way that alters its emotional impact; 6 items) and *Expressive Suppression* (inhibiting behavioral responses to an emotion-arousing event; 4 items). All items are rated on a 7-point Likert scale (1 = ‘strongly disagree’ to 7 = ‘strongly agree’), with higher scores indicating a greater use. Satisfactory internal consistency was obtained for the two emotion regulation strategies in the current sample (*Cognitive Reappraisal*: .65; *Expressive Suppression*: .66).

##### 2.2.3.3 Emotional processing styles/deficits

The Hindi version of Emotional Processing Scale (EPS-25-H) [33], a 25-item measure of emotional processing styles and deficits, was used. It has five-factors: (i) *Suppression* (excessive control of emotional experience and expression; 5 items), (ii) *Signs of Unprocessed Emotions* (intrusive and persistent emotional experiences), (iii) *Unregulated Emotions* (inability to control one's emotions; 5 items), (iv) *Avoidance* (avoidance of negative emotional triggers), and (5) *Impoverished Emotional Experiences* (detachment from and lack of insight into one's emotions). Respondents indicate their extent of agreement with the 25 statements using a 10-point Likert scale [Completely disagree (0) to Completely agree (9)]. Higher scores indicate greater emotional processing deficit. The scale had acceptable internal consistency in the present sample (Full scale: .92; Subscales: ranging from .60 to .81).

##### 2.2.3.4 Negative affect repair strategies

The Hindi version of Negative Affect Repair Questionnaire (NARQ-H) [34], a 24-item measure, was used to assess the four types of NA repair strategies: (i) *Cognitive Regulation Strategies* (changing one's thinking about emotions; 8 items), (ii) *Calming and Distractive Strategies* (relaxing, distracting or accepting negative emotions; 6 items), (iii) *Social Regulation Strategies* (either controlling feelings at outcome level or seeking help; 5 items), and (iv) *Externalizing Strategies* (e.g., self-harm, drug use; 5 items). It showed acceptable internal consistency in the present sample for all subscales (.61–.81) except *Social Regulation Strategies* (.24).

##### 2.2.3.5 Mood regulation expectancies

The Hindi version of Negative Mood Regulation Expectancies Scale (NMRES-H) [35] consisting of 30-items was used to measure the generalized expectancy that some thought/behavior will reduce/alleviate a negative emotional state, or bring about a positive one. Items are rated on a 5-point scale (‘strongly disagree’ to ‘strongly agree’). Higher scores indicate higher expectancy of regulating one's negative mood. The internal consistency of this scale in the current sample was .82.

##### 2.2.3.6 Positive and negative affect

Participant's general level of positive and negative affectivity was assessed using the Hindi version of the Positive and Negative Affect Schedule (PANAS-H) [36]. It comprises 10 positive and 10 negative mood adjectives and the respondents indicate on a 5-point Likert scale (1 = ‘a little bit or never’ to 5 = ‘nearly always’) how frequently they experience these moods. The internal consistency of PANAS-H in the current sample was .85 for *Positive Affect* and .83 for *Negative Affect.*

### 2.3 General procedure

All self-report questionnaires were administered in a single session, with brief breaks as per participants’ preference, in the presence of a researcher.

### 2.4 Data analyses

All analyses, unless specified otherwise, were performed using SPSS for Windows (version 25), with alpha level for significance testing set at <.05. No serious deviations from normality (tested using Kolmogorov-Smirnov and Shapiro-Wilks tests) were found and the skewness and kurtosis values were also within the acceptable range [37].

Correlational analyses (Pearson's *r*) were conducted to examine the relationship between the DM facets and specific symptom dimensions, and the three global distress indices (PST, PSDI, GSI) on the SCL 90-R-H. Correlational analyses were also used to examine the relationship between DM facets and different emotional constructs, as well as between symptom dimensions and emotional constructs. Bayesian correlation analyses that overcome the sampling-related weaknesses [38] and help to examine whether a non-significant correlation suggests absence of the theorized correlation or indicates insensitivity of data to capture the theorized association [39] were also performed. The Bayes Factor (BF_10_) was computed using JASP [40]. A BF_10_ value greater than 3 is considered substantial evidence to support the alternative hypothesis (the variables are correlated), less than .33 as evidence supporting the null hypothesis (the variables are uncorrelated), and a value between 1 and 3 as weak evidence for the alternate hypothesis [39].

A principal component analysis (PCA) with varimax rotation was carried out on all emotional constructs with adaptive or maladaptive dimensions (see Table 2, column l), except PA and NA (since they represent outcomes of the adaptive or maladaptive emotional processes/regulation strategies), to reduce the emotional constructs into a few explanatory factors.

**Table 1:** Correlations (Pearson's r) between dispositional mindfulness facets and different emotional constructs along with associated Bayes Factor (BF_10)_.

Emotional constructs		Describe r BF_10_	Acting with Awareness r BF_10_	Non-judging r BF_10_	Non-Reactivity r BF_10_	Mindfulness Total r BF_10_
**Range and differentiation of emotional experiences**	Range^a^	.36**	.11	.17**	.18**	.29**
		1.909 × 10^+6^	0.326	3.186	4.386	4990.178
	Differentiation^a^	.46**	.16*	−.06	.36**	.31**
		1.493 × 10^+12^	1.951	0.133	2.110 × 10^+6^	21797.203
**Use of emotion regulation strategies**	Cognitive reappraisal^a^	.07	.07	−.11	.25**	.08
		0.14	0.154	0.364	300.018	0.171
	Expressive suppression^b^	−.30**	−.29**	−.29**	−.01	−.35**
		7374.181	5142.506	3651.534	0.079	797311.65
**Emotion processing deficits**	Suppression^b^	−.31**	−.40**	−.28**	−.14^#1^	−.42**
		19468.941	2.721 × 10^+8^	1646.278	0.787	6.629 × 10^+9^
	Unregulated emotions^b^	−.27**	−.41**	−.40**	−.15^#2^	−.47**
		1030.61	1.004 × 10^+9^	4.372 × 10^+8^	1.394	3.602 × 10^+12^
	Impoverished emotional experiences^b^	−.31**	−.48**	−.36**	−.20**	−.50**
		31700.458	7.967 × 10^+12^	2.837 × 10^+6^	13.642	1.952 × 10^+15^
	Signs of unprocessed emotions^b^	−30**	−.36**	−.47**	−.11	−.48**
		10373.9	2.373 × 10^+6^	6.676 × 10^+12^	0.35	7.954 × 10^+12^
	Avoidance^b^	−.36**	−.47**	−.35**	−.12^#3^	−.50**
		4.936 × 10^+6^	1.837 × 10^+12^	774976	0.556	4.101 × 10^+14^
	Emotion processing deficits - Total^b^	−.37**	−.50**	−.44**	−.17**	−.56**
		6.783 × 10^+6^	6.148 × 10^+14^	3.014 × 10^+10^	3.455	6.647 × 10^+19^
**Negative affect repair strategies**	Cognitive regulation strategies^a^	.17**	.07	−.16^#4^	.24**	.09
		2.63	0.138	2.097	101.94	0.212
	Calming & distractive strategies^a^	.24**	.06	**.02**	.25**	.19**
		112.309	0.127	0.081	283.131	6.823
	Social regulation strategies^a^	.15^#5^	.12	−.02	−.02 0.084	.09
		1.201	0.476	0.084		0.218
	Externalizing strategies^b^	−.29**	−.39**	−.24**	−.04	−.38**
		4224.949	6.087 × 10 + 7	191.839	0.092	1.649 × 10 + 7
**Negative mood regulation expectancies**	Negative mood regulation expectancies^a^	.48**	.41**	.13^#6^	.21**	.46**
		3.402 × 10 + 13	1.257 × 10 + 9	0.641	22.016	7.428 × 10 + 11
**Positive and negative affectivity**	Positive affect^a^	.42**	.26**	.13^#7^	.31**	.40**
		5.150 × 10 + 9	750.984	0.724	16931.04	2.953 × 10 + 8
	Negative affect^b^	−.38**	−.42**	−.32**	−.19**	−.50**
		4.605 × 10 + 7	5.805 × 10 + 9	107997.3	8.093	3.653 × 10 + 14

*p = .011, **p < .001, ^#1^*p* = .031, ^#2^*p* = .016, ^#3^*p* = .047, ^#4^*p* = .010, ^#5^*p* = .019, ^#6^*p* = .040, ^#7^*p* = .034.

a = Adaptive dimensions.

b = Maladaptive dimensions.

**Table 2:** The rotated component matrix of various dimensions of emotional constructs.

	Component 1: Maladaptive emotion processing style	Component 2: Adaptive emotion processing style
Range		.43
Differentiation		.72
Cognitive regulation strategies		.75
Calming & distractive strategies		.67
Negative mood regulation expectancy	−.56	.47
Cognitive reappraisal		.57
Expressive suppression	.50	
Suppression	.80	
Unregulated emotions	.81	
Impoverished emotional experiences	.86	
Signs of unprocessed emotions	.77	
Avoidance	.85	
Externalizing strategies	.53	
**% variance explained**	31.12	17.80

**Fig. 1:**
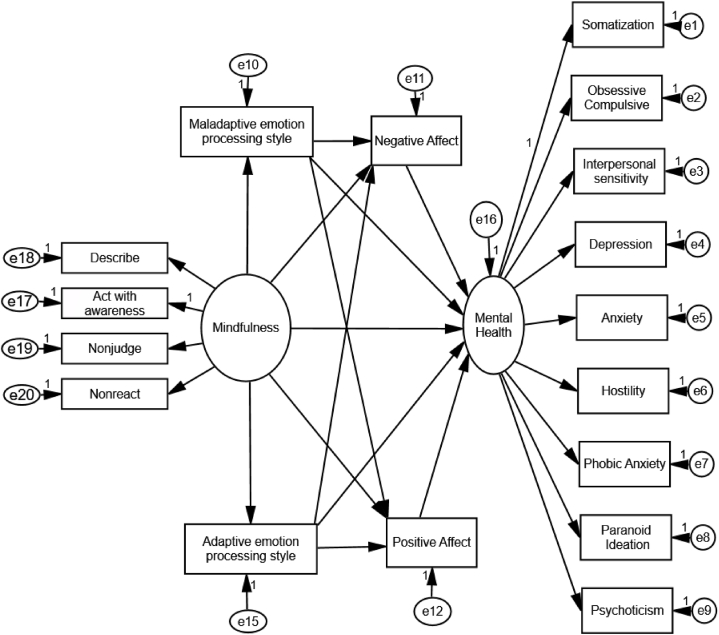
The structural model representing the hypothesized direct and indirect (emotion-mediated) pathways linking dispositional mindfulness to mental health problems (as indexed by the Global Severity Index of the SCL-90-R).

We then tested a structural model with DM as the latent construct interacting with adaptive and maladaptive emotional processing styles directly or indirectly in predicting the levels of positive and negative affectivity, which in turn predict mental health (a latent construct). To test the proposed model in AMOS 24, the standard two steps of applying SEM were followed: (1) estimating the goodness of fit of the measurement model, which specifies the number of factors, how various indicators are related to the factors, and the relationships among indicator errors; and (2) testing the structural model, which specifies how various factors are related to one another (e.g., direct or indirect effects, no relationship). Thus, we first tested the measurement models of DM (using its four facets as indicator variables) and mental health (using the nine subscales of the SCL-90-R as indicator variables). This was followed by testing of the structural model (Fig. 1) based on the conceptual framework and empirical evidence as outlined in the introduction.

The goodness-of-fit of measurement and structural models was assessed as per the criteria [41]: (a) the ratio of maximum-likelihood chi-square to the degrees of freedom (χ^2^/df) < 5, (b) Adjusted Goodness-of-Fit Index (AGFI) > .90, (c) Bentler's Comparative Fit Index (CFI) > .95, (d) Root-Mean-Square Error of Approximation (RMSEA) <0.07, and (e) Standardized Root Mean Square Residual (SRMR) < .08. We not only tested the global fit of the SEM model based on the various goodness-of-fit indices but also relied on the local fit of the model based on testing the significance of the direct and indirect path coefficients using the conventional *p* value and bias-corrected 95 % Bootstrap confidence interval.

To rule out a potential confounding effect of sex in the DM – mental health relationship, we performed a moderated regression analysis after mean centering the continuous predictors. Similarly, to rule out the possibility that observed mediating role of emotional constructs (if any) is not due to a potential overlap between the emotional constructs (mediators) and mental health (outcome variable), we conducted a principal component analysis (PCA) of all the mediator variables (viz., emotional range and differentiation, cognitive reappraisal, expressive suppression, signs of unprocessed emotions, unregulated emotions, suppression, avoidance, impoverished emotional experiences, cognitive regulation strategies, calming and distractive strategies, and externalizing strategies) and the nine subscales of SCL-90-R (indicator of the latent construct of mental health).

Lastly, as further testing of the prediction that the beneficial mental health effects of DM are largely explained by the reduction of maladaptive emotion processing style, the relative contribution of DM to mental health was explored after controlling for the effects of adaptive and maladaptive emotion processing styles (see Results). For this purpose, a hierarchical regression analysis was conducted taking maladaptive emotion processing style in the first block of predictors, DM facets in the second block of predictors, and GSI as the criterion. Similarly, to explore the contribution of DM facets after controlling for the adaptive emotion processing style in predicting mental health, a hierarchical regression analysis was conducted taking adaptive emotion processing style in the first block and DM facets in the second block of predictors.

## 3 Results

### 3.1 Self-report measures

The descriptive statistics for self-report measures (Supplementary Table 2) showed a wide range of scores with acceptable variance (acceptable Mean-to-SD ratio), confirming their suitability for dimensional analyses.

### 3.2 Dispositional mindfulness, mental health, and emotional constructs

The *Describing*, *Acting with Awareness*, *Non-judging*, and total mindfulness (FFMQ-H) scores correlated significantly negatively (mostly moderate) with specific measures of mental health as well as the three global distress indices (PST, PSDI, and GSI) assessed by the SCL-90-R-H (Supplementary Table 3). The *Non-reactivity* facet also correlated negatively with symptoms though the correlations for *Somatization*, *Anxiety*, *Psychoticism*, and *PSDI* failed to reach significance (Supplementary Table 3). All significant correlations that were found to be significant using the frequentist approach also received strong support from the Bayesian analysis as the Bayes Factors (BF_10_) were much higher than the minimum required value of 3 (Supplementary Table 3), except for the correlations of the *Non-reactivity* facet with the global distress indices and specific symptom domains (as all BFs were below 3) with the exception of obsessive-compulsive and interpersonal sensitivity domains (BF_10_ = 13.37 and 6.58, respectively).

Most mindfulness facets correlated negatively (mostly moderately) with maladaptive emotion regulation dimensions (Expressive Suppression*;* ERQ-H)*,* emotion processing (*Suppression, Unregulated Emotion, Impoverished Emotional Experience, Signs of Unprocessed Emotions, Avoidance*; EPS-25-H)*,* negative affect repair strategy (Externalizing Strategies, NARQ-H)*,* and NA (PANAS) (Table 1). Conversely, most mindfulness facets had small-to-moderate positive correlations with adaptive dimensions of *Range* and *Differentiation* of Emotional Experiences (RDEES-H)*, Calming & Distractive Strategies* (NARQ-H) to repair mood, *Negative Mood Regulation Expectancies (NMRES-H)*, and *PA.* Only the *Describing* facet of DM showed a (very small) positive association with *Social Regulation Strategies* (NARQ-H) to repair *NA*, while the relationships with *Cognitive Reappraisal* (ERQ-H) to regulate emotions and *Cognitive Regulation Strategies* (NARQ-H) to repair mood were inconsistent across different DM facets. Bayesian correlation analysis strongly supported these findings except for a few pairs where the BF_10_ values ranged between 1 and 3 (correlations: *Describing* with *Cognitive Regulation* and *Social Regulation;* *Acting with Awareness* with *Differentiation*; *Non-judging* with *Cognitive Regulation and* *PA*; *Non-Reactivity* with *Unregulated Emotions*) offering only a weak support (Table 1).

Lastly, mental health problems and the global indices on the SCL-90-R-H had small-to-moderate positive correlations with all maladaptive emotion regulation dimensions (Supplementary Table 3)*,* and small-to-moderate negative correlations with three of the adaptive dimensions, namely, *Differentiation* of Emotional Experiences (RDEES-H), *Negative Mood Repair Expectancies (NMRES-H),* and *PA* (Supplementary Table 3). Mental health and distress variables had very low negative correlations with adaptive dimensions of *Range* of Emotional Experiences (RDEES-H), and *Calming* and *Distracting* and *Social Regulation Strategies* (NARQ-H); mostly non-significant negative correlations with *Range* of Emotional Experiences (RDEES-H); and no significant correlations with *Cognitive Reappraisal* (ERQ-H) and *Cognitive Regulation Strategies* (NARQ-H). Bayesian correlation analysis strongly supported the observed significant correlations except a few pairs of correlations, for which a weak support was obtained as BF10 ranged between 1 and 3 (Supplementary Table 4). There were a few correlations, mostly the correlation of *Social Regulation Strategies* with SCL-90-R subscales, where this analysis supported the null hypothesis (the variables are uncorrelated).

### 3.3 Summarizing emotional constructs

The PCA yielded four factors with eigen values > 1, but the scree plot suggested two factors which (together) explained 48.92 % of the total variance (Table 2). *Emotional Processing Deficits*, *Expressive Suppression*, *Externalizing Strategies,* and *Negative Mood Regulation Expectancy* loaded positively and significantly on the first factor. We labelled it ‘maladaptive emotion processing style’. *Range* and *Differentiation* of *Emotional Experiences*, *Cognitive Reappraisal*, *Cognitive Strategies* as well as *Calming & Distractive Strategies* of *Negative Affect Repair* and *Negative Mood Regulation Expectancy* loaded significantly and positively on the second factor, which we labelled ‘adaptive emotion processing style’. *Social Regulation Strategy* was eliminated (with very low and non-significant loadings on both factors).

### 3.4 Roles of adaptive and maladaptive emotion processing styles in mindfulness-mental health relationship

**Fig. 2:**
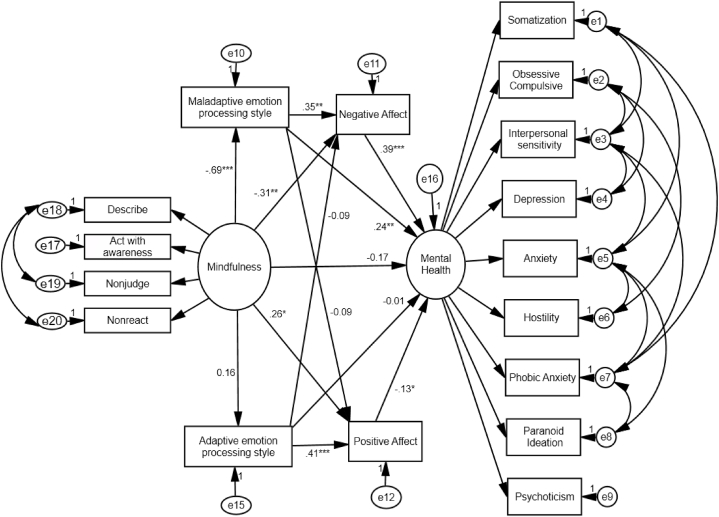
The structural model showing the interplay between dispositional mindfulness and emotional factors in predicting mental health problems.

The test of hypothesized structural model (Fig. 1), with DM (represented by a measurement model) as a predictor, emotional factors as mediators, and mental health as an outcome variable (represented by a measurement model), yielded an excellent fit to the data as almost all of the goodness-of-fit indices (χ^2^/df = 2.57, GFI = .90, AGFI = .844, TLI = .94, CFI = .96, RMSEA = .078, SRMR = 0.058) were well above the recommended cut-off [41]. The standardized estimates for each of the paths are displayed in Fig. 2. This model revealed that DM significantly and directly predicted maladaptive emotion processing style as well as NA and PA (β = −.69, −.31, .26 respectively; *p* = .001, *p* = .007 & *p* = .022 respectively). The direct relationship between DM and mental health though failed to reach the conventional level of significance, a trend level effect was noted (β = −.17; *p* = .095). Maladaptive emotion processing style significantly predicted mental health and NA (β = .24, & .35, respectively; *p* = .007 & *p* = .004, respectively). No significant relationship was observed between maladaptive emotion processing style and PA (β = −.09; *p* = .31). Adaptive emotion processing style significantly predicted *PA* **(**β = .41; *p* = .001) and its trend level effect was noted on *NA* **(**β = −.086; *p* = .074). No substantial relationship was noted between adaptive emotion processing style and mental health **(**β = −.01; *p* = .88).

DM predicted mental health through multiple pathways combining adaptive and maladaptive emotion processing styles as well as PA *and* NA (Table 3). The magnitudes of standardized path coefficients suggest that pathways involving maladaptive emotion processing style and NA, maladaptive emotion processing style alone, and NA were the best intermediary pathways between DM and better mental health. On the contrary, pathways involving adaptive emotion processing style and PA explained only a small amount of variance. The indirect effect of DM via adaptive emotion processing as well as via adaptive emotion processing and NA or maladaptive emotion processing and PA were statistically non-significant. Furthermore, the total indirect effect of DM via maladaptive emotion processing and NA was stronger **(**β = −.3; *p* = .001) compared to adaptive emotion processing and PA **(**β = −.04; *p* = .013).

**Table 3:** Mediating paths between mindfulness and mental health (lower scores on latent construct of mental health indicate better mental health) and total indirect effects of mindfulness on mental health.

Mediating paths between mindfulness and mental health problems and pooled indirect effects through different pathways	Path coefficients (β)	95% bias corrected Confidence Interval		*p*-value
		Lower	Upper	
**Adaptive emotion processing style × PA**	−.01*	**−.03**	**.00**	**.04**
Adaptive emotion processing style	−.00	−.02	.01	.73
**PA**	−.03*	**−.09**	**−.005**	**.02**
Adaptive emotion processing style × NA	−.01	−.02	.00	.06
**Maladaptive emotion processing style × NA**	−.10**	**−.17**	**−.03**	**.002**
**Maladaptive emotion processing style**	−.15**	**−.26**	**−.06**	**.004**
**NA**	−.11*	**−.20**	**−.04**	**.004**
Maladaptive emotion processing style × PA	−.01	−.03	.00	.195
**Total indirect effect through maladaptive emotion processing style and NA**	−.35***	**−.51**	**−.22**	**.001**
**Total indirect effect through adaptive emotion processing style and PA**	−.04*	**−.12**	**−.01**	**.013**
**Total indirect effect**	−.39***	**−.60**	**−.26**	**.001**

PA=Positive Affect; NA=Negative Affect; *p < .05, **p < .01, ***p < .001.

As a confirmatory test of the above finding, hierarchical regression analysis with maladaptive emotion processing style entered in block 1 (and DM in block 2) explained 36.2 % of the variance, with DM explaining only an additional 4.5 % of the variance (though significant) in the mental health levels (Table 4). When the analysis was repeated with adaptive emotion processing style, it explained only 2.4 % variance in addition to DM (which uniquely explained 25.3 % of the variance) in GSI (Table 4).

**Table 4:** Results of hierarchical regression analyses using facets of dispositional mindfulness as predictors and the global severity index as criterion after controlling for (A) maladaptive and (B) adaptive emotion processing styles.

	Predictors	R	R^2^	R^2^ Change	F Change	*p*-value	β	*t*	*p*-value
**A**	Step1: Maladaptive emotion processing style	.60	.36	.36	144.26	<.001	.60	12.01	<.001
	Step 2: Facets of dispositional mindfulness^a^	.64	.41	.04	19.12	<.001	−.26	−4.37	<.001
**B**	Step 1: Adaptive emotion processing style	.16	.02	.02	6.27	.013	−.16	−2.51	.013
	Step 2: Facets of dispositional mindfulness^a^	.53	.28	.25	88.60	<.001	−.52	−9.41	<.001

a *Describing*, *Acting with Awareness*, *Non-judging*, *Non-reactivity*.

The PCA of mediators and outcome variables yielded three distinct components (based on the scree plot). Component 1 loaded on mental health variables (SCL-90-R-H subscales), component 2 loaded on variables defining the maladaptive emotion processing styles, and component 3 on the adaptive emotion processing styles (Table 5). The only exception was *Externalizing Strategies*, which loaded on the first component of mental health variables. Observed non-overlap between mediators and outcome variables coupled with a non-significant moderating effect of sex in the DM–mental health relationship (β = .008, *p* = .098) suggest that observed direct and indirect effects of DM on mental health may be attributed to the constituent processes of DM and/or contingent emotional processing styles, PA and NA.

**Table 5:** The rotated component matrix of mediator (emotional constructs) and outcome (mental health) variables.

	Component 1 Mental health problems	Component 2 Maladaptive emotion processing style	Component 3 Adaptive emotion processing style
Anxiety	.896		
Psychoticism	.896		
Depression	.872		
Interpersonal sensitivity	.862		
Somatization	.855		
Paranoid Ideation	.843		
Phobic Anxiety	.811		
Obsessive-Compulsive	.804		
Hostility	.785		
Externalizing strategies	.522		
NMRES Total			
Suppression		.776	
Signs of unprocessed emotions		.766	
Impoverished emotional experiences		.749	
Avoidance		.730	
Unregulated emotions		.724	
Expressive suppression		.566	
Cognitive regulation strategies			.744
Differentiation			.731
Calming/distractive strategies			.657
Reappraisal			.585
Range			.480

## 4 Discussion

Addressing the first aim of the study, the findings showed higher DM to be (a) associated with relatively better mental health and psychological well-being, and (b) positively associated with adaptive dimensions (a greater range and differentiation of emotional experiences, more use of calming and distraction strategies to repair mood, higher levels of negative mood regulation expectancy, and PA) and negatively associated with maladaptive dimensions (less emotional processing deficits, less NA, less use of externalizing strategies to repair NA/mood, and expressive suppression to regulate emotions) of various emotional constructs. Our finding of relatively better mental health and psychological well-being in naturally-mindful people (i.e., with higher DM scores) is in line with our hypothesis and consistent with previous studies [10,42]. The findings showing DM to correlate positively with adaptive, and negatively with maladaptive dimensions of various emotional constructs suggest that naturally-mindful people are more likely to accept and recover from unpleasant emotional experiences and *NA*, and less likely to have poor emotional insight, intrusive emotional experiences, or unregulated emotions [42]. Our findings are consistent with earlier findings showing that elevated mindfulness is associated with heightened emotional awareness [43], emotion recognition and labeling [44], as well as meta-cognitive insight [24]. Interestingly, our findings also hinted towards a somewhat differential pattern of association of DM facets with various strategies to repair *NA*. While most DM facets correlated positively with *Calming and Distraction* (adaptive) and negatively with *Externalizing strategies* (maladaptive), they did not correlate with *Social Regulation* strategies. However, the *Social Regulation* (and no other) sub-scale had poor internal consistency in this sample (see 2.2.3.4 *Negative Affect Repair Strategies*), thus findings need to be treated with caution. Regardless, DM may be associated more consistently with calming strategies because mindfulness involves metacognitive awareness over inner experiences as well as calming and decentering in the face of overwhelming emotions [29]. Overall, our findings support earlier findings showing that mindful awareness promotes healthy engagement with emotions [45], and enhances authentic emotional experiences and expressions [46].

Concerning the study's second aim, we found that while DM is linked with greater PA, reduced NA, and better mental health, the mechanism through which psychological health is enhanced works by reducing maladaptive emotion processing. Specifically, the pathways involving maladaptive emotion processing style and NA, maladaptive emotion processing style alone, as well as NA emerged as the best intermediary pathways between DM and mental health. On the contrary, pathways involving adaptive emotion processing style and PA explained only a small amount of variance. The findings from the hierarchical regression analyses further supported this interpretation in showing that maladaptive emotion processing style explained a much larger portion of mindfulness-mental health covariance compared to adaptive emotion processing style. However, mindfulness in itself is considered an adaptive emotion regulation strategy [42], and may well work through some other mechanisms that were not captured by the ‘adaptive emotion processing style’ factor in our study. A further issue deserving acknowledgment is that the study findings might be true only for a young homogenous Indian student population leading an active college/university life, and may or may not generalize to community samples and other age groups in India or other countries and cultures.

To our knowledge, ours is the most comprehensive study with a comparable focus on both negative and positive emotional pathways as possible mediators of mindfulness-mental health relationship, which showed that mindfulness is associated with better mental health chiefly through reduced NA, with little influence of PA. Similarly, Creswell and Lindsay [47] showed that a significant pathway linking mindfulness training with better physical health is reduced distress, while presence of PA is likely to be the second important pathway which promotes physical health independently [48]. These and our findings are perhaps not surprising given that both Buddhist philosophy and its techniques, which contributed to the development of traditional mindfulness meditation [49], are rooted in eradicating or reducing suffering by removing negative cognitive-affective experiences rather than directly promoting positive psychological resources, though in experienced mindfulness practitioners affect may become more positive effortlessly.

Our findings may have important implications for self-management of psychological well-being. Given the ever-increasing demand for mental health services globally, a proactive and scalable approach to self-management of mental health is much needed [42]. The present findings suggesting better mental health via reduction in negative/maladaptive emotion processing styles, as well as earlier findings of enhancement in DM through training in mindfulness meditation [50], encourage psychological interventions/meditation training to improve mindfulness, particularly in individuals with low DM, with a targeted focus on maladaptive emotion processing styles. Even brief meditation training has been found to be effective in reducing NA not only in the meditation practitioners [51] but also in their non-meditating partners [52], so there may be wide-ranging societal benefits of such initiatives.

## 5 Limitations

First, we used a correlational design, and therefore the findings cannot speak to the question of causality in the DM-mental health relationship. Second, our findings are based on self-report measures, and should be further examined using reliable behavioral, psychophysiological and/or neural markers of mindfulness and relevant emotional constructs. Third, our findings are based on an opportunistic sample of young, educated students who may be better equipped to deal with their negative emotions and protect mental health as earlier studies have noted that young students, especially females, in higher education generally report a good level of resilience [53]. This limits the generalizability of our findings beyond the studied population. Furthermore, the nature of mental health issues in educated young adults and various resources available to them may differ from that of less educated (non-educated) young adults, middle-aged adults or older populations. Future studies with larger and heterogenous samples using objective and experimental markers of various emotion processing styles are needed to validate the present findings.

## 6 Conclusions

The present findings indicate that reduced maladaptive emotion processing style and decreased NA mediate DM-mental health relationship, but further research is needed to establish a causal link between these factors. The findings encourage developing and promoting self-help psychological interventions for increasing mindfulness, especially in individuals who are low on DM, targeting maladaptive emotion processing to reduce mental health concerns and distress.

## Funding statement

The research was supported by the Bial Foundation (92/18; awarded to VK and RP) and the Indian Council of Medical Research (3/1/3-ICMR-JRF/2011/HRD/12/81003; awarded to SPM).

## Data availability statement

The data supporting this research are available in Open Science Framework (OSF) and can be accessed using the link: https://osf.io/xk9yc/?view_only=3cb854e50ea741d89eb8762e72b63257.

## CRediT authorship contribution statement

**Rakesh Pandey:** Writing – review & editing, Writing – original draft, Visualization, Supervision, Project administration, Methodology, Investigation, Funding acquisition, Formal analysis, Conceptualization. **Satchit Prasun Mandal:** Writing – original draft, Visualization, Project administration, Methodology, Investigation, Funding acquisition, Formal analysis, Data curation, Conceptualization. **Meenakshi Shukla:** Writing – review & editing, Writing – original draft, Visualization. **Vishnukant Tripathi:** Methodology, Data curation. **Elena Antonova:** Writing – review & editing, Writing – original draft. **Veena Kumari:** Conceptualization, Funding acquisition, Writing – original draft, Writing – review & editing.

## Declaration of competing interest

The authors declare that they have no known competing financial interests or personal relationships that could have appeared to influence the work reported in this paper.
