# Supplementary material for: Attenuated maladaptive emotion processing as a potential mediator of the relationship between dispositional mindfulness and mental health
Source: Heliyon. 2023 Nov 2;9(11):e21934. doi: 10.1016/j.heliyon.2023.e21934 (PMC10658320; doi:10.1016/j.heliyon.2023.e21934)
Supplement: Multimedia component 1 [file mmc1.docx]

**Supplementary Materials**

**Supplementary Table 1**

*Additional details of the self-report measures*

| Hindi adaptation of Five-Facet Mindfulness Questionnaire (FFMQ-H; Mandal et al., 2017),  originally developed (in English) by Baer et al. (2006) | The internal consistency reliability of FFMQ-H full scale (.85) and its facets (*Describing*= .79; *Acting with Awareness*= .81; *Non-judging*= .75; *Non-reactivity*= .61) are good-to-excellent (Mandal et al., 2017). |
| --- | --- |
| Hindi adaptation of the 90-item Revised Symptom Checklist (SCL-90-R-H; Dubey & Pandey, 2011),  originally developed (in English) by Derogatis (1977) | SCL-90-R has good internal consistency and test-retest reliability (Derogatis, 1994). |
| Hindi adaptation of Range and Differentiation of Emotional Experiences (RDEES-H; Mandal et al., 2017), (RDEES-H; Mandal et al., 2017), originally developed (in English) by Kang & Shaver (2004) | The RDEES-H has adequate internal consistency as reported previously (Cronbach’s alpha coefficient: overall .81; *Range*: .67; *Differentiation*: .82; Mandal et al., 2017). |
| Hindi adaptation of Emotion Regulation Strategies (ERQ-H; Khetrapal et al., 2007), originally developed (in English) by Kang & Shaver (2004) | The Hindi version of ERQ (ERQ-H) has acceptable reliability (Cronbach’s alpha coefficient: *Cognitive Reappraisal*: .58; *Expressive Suppression*: .40) and validity (Khetrapal et al., 2007). |
| Hindi adaptation of Emotional Processing Styles/Deficits (EPS-25; Dubey & Pandey, 2012), originally developed (in English) by Gross & John (2003) | The EPS-25-H has a good concurrent and convergent validity as well as a satisfactory internal consistency (.90) and test–retest reliability (.80) for the overall scale, and for the majority of the subscales (Dubey & Pandey, 2012). |
| Hindi adaptation of Negative Affect Repair Strategies (NARQ-H; Dubey and Pandey, 2013), originally developed (in English) by Eberle (2009) | The NARQ-H yields an adequate internal consistency for the four strategy subscales (.79 to .82). |
| Hindi adaptation of Negative Mood Regulation Expectancies (NMRES-H; Dubey et al., 2013), originally developed (in English) by Catarizaro & Mearns (1990) | All items begin with the same stem: "When I am upset I believe that....". Examples of items include "I can usually find some way to help myself feel better" and "Telling myself it will pass will help me calm down". The NMRES-H has good reliability (Cronbach’s alpha=.81) and validity (Dubey et al., 2013). |
| Hindi adaptation of Positive and Negative Affect (PANAS-H; Pandey & Srivastava, 2008), originally developed (in English) by Watson et al. (1988) | The PANAS-H has been found to be reliable (internal consistency for *Positive Affect:*.80; *Negative Affect:* .78) and valid (Pandey & Srivastava, 2008). |

**References** (additional to those cited the main text)

Baer, R. A., Smith, G. T., Hopkins, J., Krietemeyer, J., & Toney, L. (2006). Using self-report assessment methods to explore facets of mindfulness. *Assessment*, *13*(1), 27–45. https://doi.org/10.1177/1073191105283504

Baker, R., Thomas, S., Thomas, P. W., & Owens, M. (2007). Development of an emotional processing scale. *Journal of Psychosomatic Research*, *62*(2), 167–178. https://doi.org/10.1016/j.jpsychores.2006.09.005

Catarizaro, S. J., & Mearns, J. (1990). Measuring generalized expectancies for negative mood regulation: initial scale development and implications. *Journal of Personality Assessment*, *54*(3–4), 546–563. https://doi.org/10.1080/00223891.1990.9674019

Derogatis, L. R. (1977). Symptoms checklist-90. *Administration, Scoring, and Procedures Manual for the Revised Version*.

Derogatis, L. R. (1994). *SCL-90-R: Administration, scoring and procedures manual. National Computer Systems*.

Eberle, N. (2009). *Negative Affect Repair Questionnaire (NARQ): development and validation of an instrument assessing negative affect regulation strategies* (Doctoral dissertation). Rheinisch-Westfälischen Technischen Hochschule Aachen.

Gross, J. J., & John, O. P. (2003). Individual Differences in Two Emotion Regulation Processes: Implications for Affect, Relationships, and Well-Being. *Journal of Personality and Social Psychology*, *85*(2), 348–362. https://doi.org/10.1037/0022-3514.85.2.348

Kang, S., & Shaver, P. R. (2004). Individual differences in emotional complexity: Their psychological implications. *Journal of Personality*, *72*(4), 687–726. https://doi.org/10.1111/j.0022-3506.2004.00277.x

Watson, D., Clark, L. A., & Tellegen, A. (1988). Development and Validation of Brief Measures of Positive and Negative Affect: The PANAS Scales. *Journal of Personality and Social Psychology*, *54*(6), 1063–1070. https://doi.org/10.1037/0022-3514.54.6.1063

**Supplementary Table 2**

*Descriptive statistics for self-report measures of mindfulness, mental health and emotional constructs*

| **Measures** | **Scales/Indices** | **Mean (SD)** | **Observed Score Range** | | **Possible Score Range** | |
| --- | --- | --- | --- | --- | --- | --- |
|  |  |  | Min | Max | Min | Max |
| **Mindfulness facets**  (FFMQ-H) | Describe | 29.36 (5.15) | 14 | 40 | 8 | 40 |
|  | Acting with Awareness | 29.36 (5.81) | 13 | 40 | 8 | 40 |
|  | Non-judging | 21.41 (5.36) | 9 | 35 | 7 | 35 |
|  | Non-reactivity | 15.39 (3.58) | 7 | 25 | 5 | 25 |
|  | Mindfulness Total | 95.52 (13.8) | 56 | 140 | 28 | 140 |
|  |  |  |  |  |  |  |
|  |  |  |  |  |  |  |
| **Mental health**  (SCL 90-R-H) | Positive Symptom Total (PST) | 43.60 (23.42) | 0 | 90 | 0 | 90 |
|  | Positive Symptom Distress Index (PSDI) | 1.54 (.45) | 1 | 3 | 1 | 4 |
|  | Global Severity Index (GSI) | .80 (.57) | 0 | 2.50 | 0 | 4 |
|  |  |  |  |  |  |  |
| **Emotional Constructs** | |  |  |  |  |  |
|  |  |  |  |  |  |  |
| **Range and differentiation of emotional experiences** (RDEES-H) | Range ^A^ | 31.06 (5.94) | 13 | 49 | 7 | 49 |
|  | Differentiation ^A^ | 31.85 (7.20) | 14 | 49 | 7 | 49 |
|  |  |  |  |  |  |  |
| **Use of emotion regulation strategies** (ERQ-H) | Cognitive reappraisal ^A^ | 27.91 (6.33) | 11 | 42 | 6 | 42 |
|  | Expressive suppression ^M^ | 15.57 (5.47) | 4 | 28 | 4 | 28 |
| **Emotion processing deficits**  (EPS-25-H)    **Negative affect repair strategies**  (NARQ-H) | Suppression ^M^ | 19.06 (9.75) | 0 | 45 | 0 | 45 |
|  | Unregulated emotion ^M^ | 22.66 (9.59) | 0 | 45 | 0 | 45 |
|  | Impoverished emotional experience ^M^ | 18.18 (9.24) | 0 | 42 | 0 | 45 |
|  | Signs of unprocessed emotions | 21.70 (7.79) | 0 | 45 | 0 | 45 |
|  | Avoidance ^M^ | 17.63 (8.70) | 0 | 41 | 0 | 45 |
|  |  |  |  |  |  |  |
|  | Cognitive regulation strategies ^A^ | 12.96 (4.26) | 0 | 26 | 0 | 32 |
|  | Calming & distractive strategies ^A^ | 8.67 (3.61) | 1 | 17 | 0 | 24 |
|  | Social regulation strategies ^A^ | 7.41 (2.49) | 0 | 14 | 0 | 20 |
|  | Externalizing strategies ^M^ | 1.35 (2.34) | 0 | 13 | 0 | 20 |
| **Negative mood regulation expectancies**  (NMRE-H) | Negative mood regulation expectancies ^A^ | 108.34 (12.81) | 70 | 137 | 30 | 150 |
|  |  |  |  |  |  |  |
| **Positive and negative affectivity**  (PANAS) | Positive Affect ^A^ | 36.88 (6.15) | 15 | 50 | 10 | 50 |
|  | Negative Affect ^M^ | 21.29 (6.56) | 10 | 43 | 10 | 50 |
|  |  |  |  |  |  |  |
|  | | | | | | |

FFMQ-H: Five Facet Mindfulness Questionnaire-Hindi version; SCL 90-R-H: 90-item Revised Symptom Checklist – Hindi version; RDEES-H: The Range and Differentiation of Emotional Experience Scale – Hindi version; ERQ-H: Emotion Regulation Questionnaire – Hindi version; EPS-25-H: The Emotional Processing Scale – Hindi version; NARQ-H: Negative Affect Repair Questionnaire – Hindi version; NMRE-H: Negative Mood Regulation Expectancies Scale – Hindi version; PANAS: Positive and Negative Affect Scale – Hindi version.

^A^ = Adaptive dimensions, ^M^ = Maladaptive dimensions

**Supplementary Table 3**

*Correlation (Pearson’s r) of various dimensions of dispositional mindfulness (FFMQ-H) with different symptom categories and three global distress indices on SCL-90-R-H and associated Byes Factor (BF_10_)* *with each correlation*

| **Mental Health** | | **Dimensions of Dispositional Mindfulness** | | | |  |
| --- | --- | --- | --- | --- | --- | --- |
| **Symptom categories** | **Describe**  **r**  **BF_10_** | | **Acting with Awareness**  **r**  **BF_10_** | **Non-**  **Judging**  **r**  **BF_10_** | **Non-reactivity**  **r**  **BF_10_** | **Mindfulness Total**  **r**  **BF_10_** |
| Somatization | -.33^**^  190041.6 | | -.42^**^  2.341×10^+9^ | -.22^**^  35.462 | -.06  .13 | -.40^**^  3.177×10^+8^ |
| Obsessive -compulsive | -.43^**^  9.357×10^+9^ | | -.50^**^  2.075×10^+14^ | -.38^**^  2.506×10^+7^ | -.20^**^  13.367 | -.57^**^  1.835×10^+20^ |
| nterpersonal sensitivity | -.38^**^  4.310×10^+7^ | | -.42^**^  3.255×10^+9^ | -.33^**^  140979.6 | -.19^#1^  6.575 | -.50^**^  2.584×10^+14^ |
| Depression | -.35^**^  1.260×10^+6^ | | -.43^**^  2.177×10^+10^ | -.29^**^  3893.436 | -.17^#2^  2.644 | -.47^**^  3.172×10^+12^ |
| Anxiety | -.37^**^  9.357×10^+6^ | | -.44^**^  8.225×10^+10^ | -.24^**^  149.839 | -.11  .323 | -.45^**^  1.263×10^+11^ |
| Hostility | -.23^**^  96.071 | | -.38^**^  2.663×10^+7^ | -.22^**^  45.002 | -.14^*^  .824 | -.37^**^  8.365×10^+6^ |
| Phobic anxiety | -.38^**^  1.606×10^+7^ | | -.34^**^  566392.4 | -.18^#1^  5.434 | -.15^#3^  1.391 | -.40^**^  1.487×10^+8^ |
| Paranoid ideation | -.28^**^  2631.527 | | -.46^**^  5.289×10^+11^ | -.32^**^  47291.55 | -.16^#4^  1.704 | -.46^**^  1.025×10^+12^ |
| Psychoticism | -.37^**^  9.809×10^+6^ | | -.48^**^  1.822×10^+13^ | -.35^**^  1.197×10^+6^ | -.11  .332 | -.51^**^  1.108×10^+15^ |
| PST | -.41^**^  7.496×10^+8^ | | -.49^**^  6.123×10^+13^ | -.32^**^  104293.2 | -.16^#4^  1.675 | -.52^**^  3.703×10^+16^ |
| PSDI | -.25^**^  182.393 | | -.39^**^  6.735×10^+7^ | -.30^**^  11485.55 | -.11  0.378 | -.41^**^  6.369×10^+8^ |
| GSI | -.40^**^  2.517×10^+8^ | | -.50^**^  2.069×10^+14^ | -.32^**^  94183.38 | -.16^#5^  1.966 | -.53^**^  3.966×10^+16^ |

***p*<.001, **p*=.03, ^#1^*p*=.003, ^#2^*p*=.008, ^#3^*p*=.016, ^#4^*p*=.013, ^#5^*p*=.011

FFMQ-H: Five Facet Mindfulness Questionnaire-Hindi version; SCL 90-R-H: 90-item Revised Symptom Checklist – Hindi version; PST: Positive Symptom Total; PSDI: Positive Symptom Distress Index (PSDI); GSI: Global Severity Index

**Supplementary Table 4**

*Correlations (Pearson’s r) between emotional constructs with different mental health problems (SCL-90-R-H) and associated Byes Factor (BF_10_)* *with each correlation*

|  | |  | | **SCL-90-R-H Mental Illness Symptom Categories** | | | | | | | | | | | |
| --- | --- | --- | --- | --- | --- | --- | --- | --- | --- | --- | --- | --- | --- | --- | --- |
|  | Emotional constructs | | Somatization  r  BF_10_ | | Obsessive Compulsive  r  BF_10_ | Inter-  personal sensitivity  r  BF_10_ | Depression  r  BF_10_ | Anxiety  r  BF_10_ | Hostility  r  BF_10_ | Phobic  Anxiety  r  BF_10_ | Paranoid  Ideation  r  BF_10_ | Psychoticism  r  BF_10_ | **PST**  **r**  **BF_10_** | **PSDI**  **r**  **BF_10_** | **GSI**  **r**  **BF_10_** |
| **Range and differentiation of emotional experiences** (RDEES-H) | Range ^A^ | | -.11  .37 | | -.11  .331 | -.11  .323 | -.02  .082 | -.12  .425 | -.06  .132 | -.16^*^  1.806 | -.04  .093 | -.10  .311 | -.14^#1^  .921 | -.02  .081 | -.10  .293 |
|  | Differentiation ^A^ | | -.19^**^  6.458 | | -.19^**^  9.105 | -.17^**^  2.835 | -.18^**^  4.775 | -.20^**^  13.171 | -.14^#2^  ~~0~~.794 | -.23^**^  83.838 | -.11  ~~0~~.385 | -.13^#3^  ~~0~~.653 | -.26^**^  512.641 | -.01  0.079 | -.20^**^  10.471 |
|  |  | | | | | | | | | | | | | | |
| **Use of emotion regulation strategies** (ERQ-H) | Cognitive reappraisal ^A^ | | -.03  .087 | | .02  .084 | -.02  .085 | .02  .083 | -.01  .079 | -.08  .194 | -.01  .08 | .00  .078 | .00  .078 | .06  .129 | -.08  .171 | -.01  .079 |
|  | Expressive suppression ^M^ | | .16^**^  2.335 | | .24^**^  157.106 | .20^**^  12.54 | .18^**^  5.979 | .22^**^  33.82 | .15^#4^  1.618 | .16^**^  2.439 | .19^**^  7.352 | .20^**^  14.523 | .20^**^  15.025 | .18^**^  3.982 | .22^**^  34.952 |
|  |  | |  | |  |  |  |  |  |  |  |  |  |  |  |
| **Emotion processing deficits** (EPS-25-H) | Suppression ^M^ | | .32^**^  56421.72 | | .40^**^  4.592×10^+8^ | .42^**^  3.069×10^+9^ | .39^**^  6.908×10^+7^ | .38^**^  1.890×10^+7^ | .39^**^  1.035×10^+8^ | .31^**^  23056.26 | .41^**^  1.065×10^+9^ | .37^**^  1.269×10^+7^ | .43^**^  1.805×10^+10^ | .32^**^  82348.38 | .43^**^  7.793×10^+9^ |
|  | Unregulated emotion ^M^ | | .34^**^  413727.6 | | .55^**^  6.696×10^+18^ | .44^**^  4.730×10^+10^ | .50^**^  4.451×10^+14^ | .42^**^  2.857×10^+9^ | .42^**^  3.364×10^+9^ | .30^**^  15166.66 | .44^**^  7.301×10^+10^ | .44^**^  9.023×10^+10^ | .45^**^  1.035×10^+11^ | .47^**^  2.450×10^+12^ | .49^**^  1.234×10^+14^ |
|  | Impoverished emotional experience ^M^ | | .38^**^  4.905×10^+7^ | | .47^**^  5.305×10^+12^ | .52^**^  7.545×10^+15^ | .47^**^  2.111×10^+12^ | .45^**^  2.025×10^+11^ | .57^**^  1.209×10^+20^ | #.38^**^  1.897×10^+7^ | .46^**^  1.136×10^+12^ | .46^**^  6.158×10^+11^ | .47^**^  5.504×10^+12^ | .47^**^  1.149×10^+12^ | .52^**^  1.969×10^+16^ |
|  | Signs of unprocessed emotions ^M^ | | .28^**^  3006.789 | | .42^**^  6.403×10^+9^ | .37^**^  1.257×10^+7^ | .34^**^  414669.6 | .34^**^  405709.4 | .30^**^  11805.93 | .31^**^  17812.81 | .32^**^  74392.14 | .34^**^  406000.9 | .39^**^  1.142×10^+8^ | .33^**^  89120.97 | .38^**^  4.540×10^+7^ |
|  | Avoidance ^M^ | | .43^**^  7.978×10^+9^ | | .51^**^  4.396×10^+15^ | .51^**^  5.192×10^+15^ | .55^**^  1.834×10^+18^ | .52^**^  5.866×10^+15^ | .47^**^  1.984×10^+12^ | .40^**^  1.605×10^+8^ | .48^**^  2.683×10^+13^ | .53^**^  5.224×10^+16^ | .54^**^  2.234×10^+17^ | .43^**^  7.723×10^+9^ | .56^**^  4.469×10^+19^ |
|  |  |  |  |  |  |  |  |  |  |  |  |  |  |  |  |
|  |  | |  | |  |  |  |  |  |  |  |  |  |  |  |
| **Negative affect repair strategies** (NARQ-H) | Cognitive regulation strategies ^A^ | | -.08  .191 | | .02  .083 | -.06  .128 | -.07  .142 | -.03  .088 | -.06  .12 | -.06  .114 | -.05  .113 | -.00  .078 | -.08  .196 | .03  .091 | -.06  .115 |
|  | Calming & distractive strategies ^A^ | | -.12^#5^  .567 | | -.10  .279 | -.16^#6^  1.659 | -.15^#7^  1.267 | -.09  .199 | -.11  .371 | -.06  .122 | -.11  .321 | -.10  .274 | -.14^#8^  .989 | -.03  .089 | -.13^#9^  .71 |
|  | Social regulation strategies ^A^ | | -.15^4^  1.54^#^3 | | -.05  .105 | -.14^#10^  .82 | -.12  .436 | -.13^#11^  .611 | -.16^#12^  2.236 | -.13^#13^  .642 | -.13^#9^  .713 | -.12  .434 | -.14^#14^  .892 | -.08  .165 | -.14^#1^  .914 |
|  | Externalizing strategies ^M^ | | .47^**^  3.480×10^+12^ | | .33^**^  154779.1 | .43^**^  9.682×10^+9^ | .48^**^  1.769×10^+13^ | .54^**^  2.457×10^+17^ | .49^**^  5.842×10^+13^ | .49^**^  4.565×10^+13^ | .41^**^  5.570×10^+8^ | .52^**^  6.006×10^+15^ | .51^**^  9.421×10^+14^ | .34^**^  378621.6 | .  52^**^  3.319×10^+16^ |
|  |  | |  | |  |  |  |  |  |  |  |  |  |  |  |
| **Negative mood regulation expectancies** (NMRE-H) | Negative mood regulation expectancies ^A^ | | -.44^**^  6.400×10^+10^ | | -.36^**^  2.416×10^+6^ | -.46^**^  8.211×10^+11^ | -.45^**^  2.682×10^+11^ | -.49^**^  1.673×10^+14^ | -.43^**^  1.892×10^+10^ | -.49^**^  4.304×10^+13^ | -.36^**^  5.298×10^+6^ | -.43^**^  1.208×10^+10^ | -.51^**^  1.656×10^+15^ | -.29^**^  5644.252 | -.49^**^  1.530×10^+14^ |
|  |  | |  | |  |  |  |  |  |  |  |  |  |  |  |
| **Positive and negative affectivity** (PANAS) | Positive affect ^A^ | | -.29^**^  5955.541 | | -.34^**^  614942.2 | -.33^**^  125742.9 | -.38^**^  1.533×10^+7^ | -.36^**^  5.235×10^+6^ | -.26^**^  589.173 | -.34^**^  291097.2 | -.28^**^  2290.239 | -.31^**^  24854.54 | -.39^**^  1.313×10^+8^ | -.19^**^  6.525 | -.36^**^  4.899×10^+6^ |
|  | Negative affect ^M^ | | .47^**^  2.780×10^+12^ | | .56^**^  4.764×10^+19^ | .58^**^  5.276×10^+21^ | .60^**^  1.477×10^+23^ | .61^**^  1.728×10^+24^ | .51^**^  1.026×10^+15^ | .49^**^  1.217×10^+14^ | .56^**^  8.449×10^+19^ | .60^**^  6.060×10^+22^ | .60^**^  5.914×10^+22^ | .50^**^  1.859×10^+14^ | .63^**^  1.491×10^+26^ |

RDEES-H: The Range and Differentiation of Emotional Experience Scale – Hindi version; ERQ-H: Emotion Regulation Questionnaire – Hindi version; EPS-25-H: The Emotional Processing Scale – Hindi version; NARQ-H: Negative Affect Repair Questionnaire – Hindi version; NMRE-H: Negative Mood Regulation Expectancies Scale – Hindi version; PANAS: Positive and Negative Affect Scale – Hindi version

^A^ = Adaptive dimensions, ^M^ = Maladaptive dimensions

***p*<.01, ^*^*p*=.012, ^#1^*p*=.026, ^#2^*p*=.031, ^#3^*p*=.039, ^#4^*p*=.014, ^#5^*p*=.046, ^#6^*p*=.013, ^#7^*p*=.018, ^#8^*p*=.024, ^#9^*p*=.035, ^#10^*p*=.030, ^#11^*p*=.042, ^#12^*p*=.009, ^#13^*p*=.040, ^#14^*p*=.027
